# A Narrative Review of the Current Knowledge on Fruit Active Aroma Using Gas Chromatography-Olfactometry (GC-O) Analysis

**DOI:** 10.3390/molecules26175181

**Published:** 2021-08-26

**Authors:** Mariana Buranelo Egea, Mirella Romanelli Vicente Bertolo, Josemar Gonçalves de Oliveira Filho, Ailton Cesar Lemes

**Affiliations:** 1 Campus Rio Verde, Goiano Federal Institute of Education, Science and Technology, Rodovia Sul Goiana, Km 01, Rural Area, Rio Verde 75901-970, GO, Brazil; 2 São Carlos Institute of Chemistry (IQSC), University of São Paulo (USP), Av. Trabalhador São-Carlense, 400, CP-780, São Carlos 13560-970, SP, Brazil; mirella.bertolo@usp.br; 3 School of Pharmaceutical Sciences, São Paulo State University (UNESP), Rodovia Araraquara-Jaú Km 1, Araraquara 14800-903, SP, Brazil; josemar.gooliver@gmail.com; 4 Department of Biochemical Engineering, School of Chemistry, Federal University of Rio de Janeiro (UFRJ), Av. Athos da Silveira Ramos, 149, Rio de Janeiro 21941-909, RJ, Brazil; ailtonlemes@eq.ufrj.br

**Keywords:** solid-phase extraction, headspace, fruit odor, trained sensory panel

## Abstract

Fruit aroma, a mixture of chemical compounds with odor, is a strong attractant derived from a complex mixture of different amounts and intensities (threshold) of chemical compounds found in fruits. The odor-producing compounds of fruit aroma are derived from carbohydrates, lipids, phenolic compounds, and mono- and sesquiterpenes, among others. The identification of compounds responsible for fruit aroma is usually conducted using gas chromatography coupled with olfactometry (GC-O). This technique separates the chemical compounds from the aroma of foods using a chromatographic column and divides the resultant outflow between the physical detector and a testing outlet (sniffing port). Trained judges describe the perceived odor in terms of the intensity of the odor zones perceived according to their training method. Moreover, the use of GC-O coupled with a mass detector (GC-MS-O) allows for the retrieval of chemical information such as identification and quantification of compounds, which can be correlated to sensory information. This review aimed to demonstrate the application of GC-MS-O in the identification of precursor compounds in fruit aroma, considering important factors for the application, main results, and most recent advances in this field.

## 1. Introduction

Fruit is botanically defined as the structure of angiosperms that develops from the ovary wall after fecundating as the enclosed seed or as seeds mature. Popularly, fruit is considered the edible part of plants that can be used in the manufacture of varied food products, including desserts, juices, and pulps, among others [1,2]. The fruit market is a sector that generates around US$ 135 billion annually and has great importance in the development, employment, and income generation of the producing regions and, mainly, as raw material for application in the various sectors of the food and beverage industries [3]. Due to the great importance of the market and the diversity of fruits, it is important to establish strict quality standards—as well as for all food products—in order to offer standardized and safe products that meet the sensory perspectives of consumers [4].

Sensory attributes such as odor and aroma are undoubtedly essential characteristics for assessing the quality of fruits, exerting a direct influence on their acceptance or refusal by consumers [5]. Thus, the determination of the flavor is an important factor for the knowledge of the specific properties of the fruits. The flavor is defined as the complex combination of olfactory, gustatory, and trigeminal sensations perceived during the tasting. It can be influenced by tactile, thermal, psychological, and/or synesthetic reactions [6]. More simply, the term flavor is defined as the sensation induced by food while being perceived by the tactile receptors in the mouth and by the senses of taste and smell [6,7]. Furthermore, this term is used to define the physiological sensation of the interaction between taste and smell (odor), resulting in the aroma, which is perceived by people during food intake.

The common characteristic of the compounds that constitute the flavor or aroma of food is their interaction with the human olfactory system, inducing specific sensations of odor. This characteristic is responsible for the chemical stimulation of the human senses that are considered decisive in food consumption [5]. Although taste and smell are both involved in the perception of these compounds, most research has been carried out for the determination of the constituent odoriferous components that constitute the food aroma [7].

In aroma research, the terms odor and odorant are recurrent and deserve a definition. The term odor refers to the perception experienced when one or more chemicals encounter the olfactory nerve. The term odorant, in turn, refers to a chemical compound (or a mixture of some of them) in the air that is part of the perception of odor [8,9]. The odor perception is considered a response to active volatile compounds that enter through the nostrils (orthonasal olfaction), while the aroma perception is the result of the sensation perceived from volatile products that enter through the mouth after chewing and swallowing, which achieve olfactory nerve receptors (retronasal olfaction) [8,10]. Thus, the characteristic aroma of foods is generally the result of the perception of dozens or hundreds of complex volatile molecules (odorants), mainly hydrophobic, found at trace levels [5,6,11,12]. In this review, we will standardize the term aroma since most of the papers reviewed consider changes that occur in the oral cavity such as mixing with saliva and increasing temperature or agitation. Although these compounds responsible for the aroma are present in extremely low concentrations, usually nanogram or picogram, they directly influence the formation of the characteristic aroma of the food and its perception by humans depends on their detection limit (threshold) [5].

The volatile compounds interact with a human receptor known as protein G, which is present in the olfactory epithelium of the nasal cavity. Once activated, the receptor triggers a sequence of events to convert the information provided by the chemical structure into a sensory stimulus [5]. For volatile compounds to reach these olfactory receptors, they usually have high vapor pressures and molecular weights, normally not greater than 300 Da [7].

Smell, from all the human senses, is the most complex and least-understood among all of them. In contrast to the physical senses (hearing, sight, and touch), smell (considered chemical sense) comprises a much larger number of receptors, of which only 900 have been studied so far [8,13]. Research in this direction is conducted considering the changes that occur during food intakes by humans, such as changes in temperatures, salivation, or chewing [5].

For the correct determination of the compounds that constitute aroma in food matrices, specialized steps of extraction and preparation of the sample are required before the instrumental analysis. Among the initial steps, there are the sampling and homogenization, followed by the preparation of the extract, cleaning, and concentration, which must be adapted and targeted to the different food matrices studied. Instrumental analysis is realized with sensitive techniques such as gas chromatography combined with an adequate detection system such as mass spectrometry (GC-MS) [14,15].

In this sense, the study of compounds responsible for food aroma must include the stages of isolation, concentration, separation, identification, and quantification of volatile chemical compounds [16,17] for which several specific techniques can be applied and that will be discussed in this review.

## 2. General Aspects of the Chromatographic Analysis of Aroma

### 2.1. Extract Preparation

Sample preparation is one of the most important steps in aroma research, which requires caution for execution. The extract must accurately characterize the aroma composition present in the food, so a representative method for the isolation of volatile compounds must include qualitative and quantitative aspects. In this context, the extracts can be prepared directly from the sample, representing the composition of the food, or they can be prepared using headspace, representing the composition of the volatile compounds released by the sample in a closed system [10].

During the sample preparation, enzymatic and non-enzymatic reactions can occur, and therefore, mild conditions are usually employed in the aroma extraction to avoid the formation of undesirable compounds [12]. In addition, additives are often added to prevent microbial growth, as well as antioxidants to decrease the speed of enzymatic reactions and oxidative processes [18,19]. Oxidation can occur in all living organisms, resulting in free radicals’ production (O_2_, OH, and H_2_O_2_), which arise naturally during metabolism and during breathing in aerobic organisms. When these radicals are produced in excess and not eliminated, they can attack the nearest molecules by subtracting electrons and starting a chain reaction where the electron-deficient molecule attacks other molecules, and so on continuously, causing damage to the compounds involved in aroma formation [20,21].

However, to mimic the process that occurs in the human body, many researchers have reported that the use of enzymatic or chemical hydrolysis in the sample preparation process can demonstrate interesting results in identifying the compounds present in the aroma. Ubeda et al. [19] using the same isolation and separation methods as Culleré et al. [22] reported the glycosylated aroma compounds of four strawberry varieties (Fuentepina, Camarosa, Candonga, and Sabrina). The samples were hydrolyzed (chemically or enzymatically) to release the precursor aroma compounds. In general, these authors observed that the longer the acid hydrolysis time, the higher the content of norisoprenoids, volatile phenols, benzenes, lactones, furaneol, and mesifurans were detected. A total of 51 compounds with aglycones were identified, 38 of which were reported for the first time. GC-O revealed furaneol, γ-decalactone, ethyl butanoate, ethyl hexanoate, ethyl 3-methyl butanoate, diacetyl, hexanoic acid, and (Z)-1,5-octadien-3-one as the most frequently modified compounds. In addition, differences were observed between the fruity, sweet, floral, and green notes of the strawberry varieties studied.

After hydrolysis and using liquid extraction of the pulp of green (*Actinidia deliciosa* “Hayward”) and golden (*Actinidia chinensis* “Hort16A”) kiwifruits, Garcia et al. [23] showed 12 and 14 odorous compounds, respectively. In this case, GC-O analysis considered the frequency and intensity (weak, medium, and strong) of the compounds, revealing the presence of 2-phenyl-ethanol and vanillin with strong intensity in both fruits, and of 2,5-dimethyl-4-hydroxy-3-(2H)-furanone (DMHF) with strong intensity and medium for green and golden kiwifruits. This study demonstrated that most compounds identified with high intensity perceived by the judges in the pulp of these fruits during GC-O were described as sweetened, which are normally found in ripe fruits. The same working group had already isolated the glycosylated precursors from the volatile compounds of kiwifruit (*Actinidia eriantha*) using adsorption on Amberlite XAD-2 resin and enzymatic hydrolysis [24]. These authors detected several bound glycosylated compounds that had not yet been reported as precursors of odorous volatile for kiwifruits, such as linoleic, linolenic, and benzoic acids and coniferyl alcohol.

Meret et al. [25] analyzed free and glycosidically bound volatiles of purees from Andean blackberry fruit (*Rubus glaucus* Benth.). For free volatiles, these authors adopted solvent extraction and SPME techniques, while bound volatiles were extracted using SPE. A total of 71 volatile compounds were identified, mostly alcohol (47.3%), including 2-heptanol (17.9%) and terpinen-4-ol (20.0%), and esters (39.8%), including ethyl and methyl benzoate (33.9% and 3.8%, respectively) as the two predominant classes. Using SPE, 53 aglycons were identified, mostly acids (57.4%), norisoprenoids (15.4%), terpenic alcohols (10.3%), and some aliphatic and shikimic alcohols (15.0%). This study demonstrates the importance of adopting different volatile extraction techniques, depending on their configuration in the food matrix.

Depending on the food matrix, sample preparation may include homogenization, centrifugation, steam distillation (SD), solvent extraction (SE), supercritical fluid extraction (SFE), pressurized fluid extraction, Soxhlet extraction, liquid–liquid extraction, and techniques involving dynamic or static headspace, such as solid-phase extraction or microextraction (SPE/SPME), among others. Many of these methods take a long time to perform and require exhaustive sample concentration steps. Ideally, the isolation of volatile compounds would comprise a single step, separating the volatile components of the matrix from the non-volatile ones, with minimal manipulation of the sample. Among the techniques that involve simplicity, automation, and cost reduction are solid-phase extraction (SPE) and solid-phase microextraction (SPME), which have been widely used over the last 20 years [15,26].

**Figure 1:**
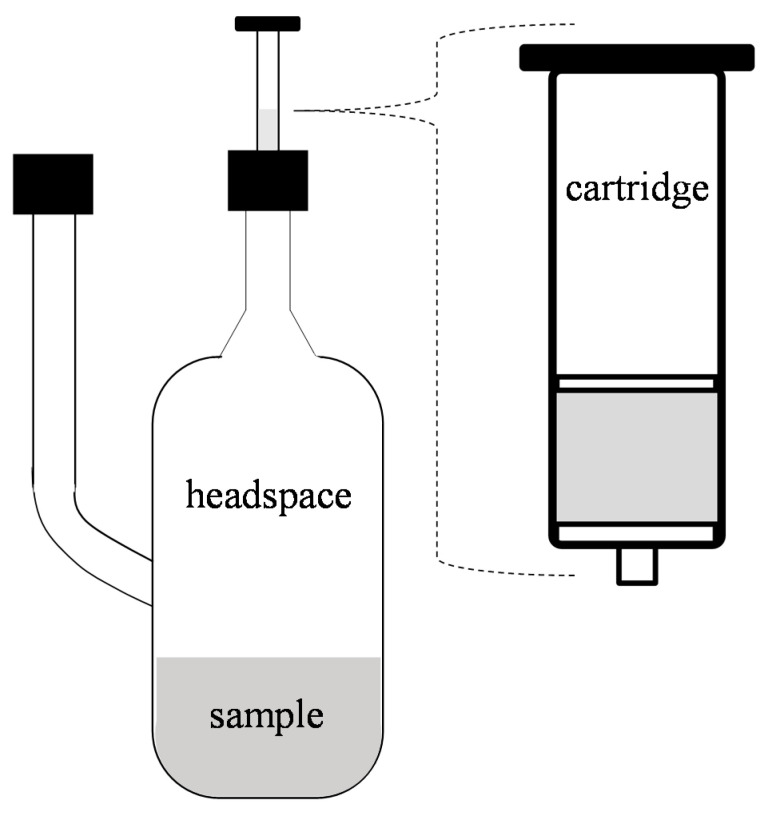
Headspace system coupled to a solid phase (SPE) with a cartridge used for the isolation of aroma compounds by the SPE method for extracting fruit pulp components. Adapted from San-Juan, Pet’Ka, Cacho, Ferreira, and Escudero [27]; Zapata et al. [15].

Among the SPE’s objectives are to reduce the level of interference, promote a high concentration factor to maximize the sensitivity of the sample, and use a solvent that is compatible with the analytical technique used. In this technique, the compounds are separated using the basic chromatography concepts, where a solid adsorbent phase performs the function of retaining the compounds, and a mobile phase (liquid, emulsified, or a gas), which is applied to elute these compounds. Figure 1 shows the simplest and most used format for SPE isolation: the system consists of a cartridge or a plastic column, usually as a syringe, containing a few milligrams or up to 10 g of packaged absorbent material [15,27].

SPE isolation has as an advantage the adequate flow distribution, which occurs when the adsorbent is efficiently packaged in the column, forming a homogeneous bed that ensures effective contact with the sample. Convenient separation of the compounds of interest is achieved when the choice of the solid phase considers the chemical nature and the polarity of the sample, as well as the solvent in contact with the sample. The pre-conditioning of the solid phase is normally carried out for cleaning and balance, achieved with the use of the solvent applied in the elution of the compounds. Finally, the sample is introduced by gravity into the column or attached to a container [15].

The SPME technique is favorable for samples that have compounds susceptible to thermal decomposition and oxidation. The basic principle of the technique is to expose a surface covered by a sorbent material (consisting of fiber-coated silica) to a sample, followed by the extraction of the compounds of interest. This stage reaches its greatest extraction when the balance between the sample and the sorption phase is reached [15]. SPME has been extensively used for its simplicity, quick sample preparation, high sensitivity, and the possibility of automation [8]. In addition, this technique uses lower amounts of solvent and samples and exploits the solid phase adsorption capacity [7,12].

The choice of the solid phase must occur by the food matrix, where the fibers used in both, SPE and SPME, can consist of divinylbenzene/carboxane/polydimethylsiloxane. The fiber must provide high efficiency in the extraction of volatile compounds and generate relevant peaks (in the area) for the studied sample. In addition, SPME can be used both, for the direct extraction of compounds that are in the liquid phase and for the extraction of compounds from headspace [7].

The headspace system, as shown in Figure 1, is a closed system where the sample is kept at a constant temperature and agitation. A flow of inert gases passes through the system to drag the aroma compounds released by the sample into the solid phase (SPE/SPME). Although the conventional headspace technique reflects a reaction of equilibrium between volatile compounds in the food and the headspace, the extract obtained in this isolation step may not reflect the same aroma perceived when the product is consumed, occurring due to the absence of the processes in the oral cavity such as breathing, dilution with saliva, and swallowing, for example, which in turn influence the release of aroma by the food and cannot be perfectly simulated in in vitro preparations [27].

Like all available techniques, the use of SPE/SPME and headspace extraction methods have some limitations. The chemical profile of the extract in this step will depend on the type, thickness, length, and quality of the selected fibers, as well as on the time, temperature, and gas flow fed to the system [5]. After trapping the volatile compounds in the solid phase, they are desorbed using a solvent so that they can be injected into the gas chromatograph [12].

### 2.2. Separation and Identification of Compounds Responsible for Food Aroma Using Gas Chromatography (GC)

About 10,000 compounds have been identified in foods, but only 3% of these compounds have a significant role in the formation of aroma, also known as active compounds [28]. Gas chromatography is an interesting method for separating, which can be associated with identification and quantification methods [12]. However, the application of this technique allows the identification of a great number of compounds, which may not be perceived by the human nose [28,29].

The importance of the active compounds in the aroma depends on the concentration in which they are found in food and on the minimum threshold perceived by the human nose (aromatic threshold). The detection of a compound in higher quantity using GC does not mean that this compound is more important for the formation of the food aroma, due to the difference in the intensity/concentration ratio of the compounds. In this way, research has been directed to the determination of compounds that significantly contribute to the formation of the aroma in foods, where gas chromatography coupled with olfactometry (GC-O) stands out as an analytical technique that associates the capillary resolution capacity with the sensitivity of the human nose [10,12].

GC-O separates the chemical compounds from the extract with a chromatographic column, followed by the division of this effluent between the physical detector and an outlet where trained judges inhale the flow (sniffing port, ODO). At the beginning of the GC-O methodology, the reproducibility was a problem since the judges reported discomfort caused by inhaling the hot and dry gas that flows from the chromatograph. This problem was solved for the combination of a humidified air effluent, thus reducing nasal discomfort [5]. The judges evaluate the effluent from the GC and make descriptive observations regarding the intensity of the aroma zones perceived in the effluent generated according to the training method. GC-O allows the researcher to know and determine the areas eluted by the chromatographic column and perceived by the judges [18,30].

**Figure 2:**
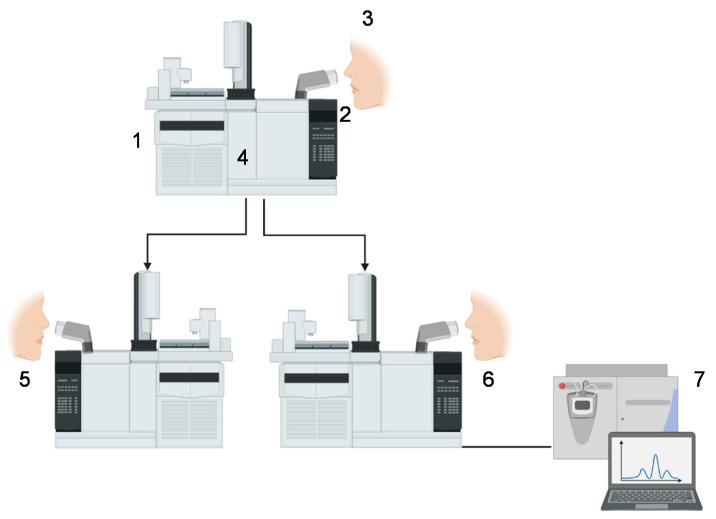
Scheme of analysis of chemical compounds to determine the aroma of fruits. 1—Injector; 2—stopwatch; 3—ODO; 4—oven; 5—ODO I; 6—ODO II; 7—mass spectrum.

Figure 2 shows the scheme of the analysis of chemical compounds to determine the fruit aroma. The concentrated extract from the aroma food extract, according to one of the methods described in item 2.1, is injected into the GC-O (Figure 2(1)) and the stopwatch is started by the judge (Figure 2(2)). The judge is positioned in front of the odorous door (ODO, Figure 2(3)), inhaling the flow separated for the chromatographic column. When the judge detects an odor (Figure 2(4)), they will write the time (accordingly with the stopwatch) and describe what and which intensity they fell, according to the training techniques described in item 3 in this review.

The compound retention time in the chromatographic column and the chromatographic column specifications together enable the calculation of the retention index of the compound separated using interpolation. For this, the separate retention time of the compound is related to the retention time of two standards (usually hydrocarbons), eluted before and after the peak of the compound of interest, respectively. In this case, the Kováts index and the linear retention index (LRI) of the compounds of interest are usually published in the literature. The Kováts index is calculated under isothermal temperature conditions and the second can be obtained when programmed temperatures are used in the column [31]. Linear retention indices are used in the identification of volatile compounds to compare the experimental elution order with the elution order described in the literature when using the same research conditions. In addition, the use of analytical standards, which help to eliminate errors, increase the reliability of compound identification [9].

In the system that contains only one GC, this equipment is coupled with a mass spectrometry detector to add information and assist the researcher in identifying the compounds. In this case, at the exit of the chromatographic column (Figure 2(4)), the flow divider will send part of the flow to the ODO and another part to a detector, e.g., the flame ionization detector (FID). However, depending on the chemical compounds present in the isolated aroma extract and their chemical nature, in some cases it is not possible to identify these compounds only by their retention index or aroma description. This is because the separation process in the chromatograph is performed according to the polarity of the compound and each separation process involves a chromatographic column with a different polarity. Thus, the aroma extract can be directed to two-dimensional chromatography that will provide more information to the researcher [32,33].

Two-dimensional gas chromatography is a system with two independent chromatographs connected with a Deans valve. The first GC usually has the same technical specifications as the one used previously (when only GC-O is used), and in the second, another GC column with a different polarity is chosen to add information about the studied aroma zone. The retention time of the zone to be identified in the first GC is previously known and from it, the system is activated to make the “cut” of the run and send this “cut” to the second chromatograph. In addition, the isolated aroma extract can be monitored using ODO I and II (Figure 2(5,6)) with trained judges in each GC. Thus, for the aroma zone studied (which may represent one or more chemical compounds), the researcher will have two linear retention indices with different polarities (polar and non-polar), their mass spectrum (detector that is normally coupled to the second GC), and odor description and intensity that were perceived in that extract by the judges [19,30,32,34]. The use of GC-O together with the detection of compounds using mass spectrum (Figure 2(7)) constitutes the basic techniques for the separation, identification, and quantification of active aromas in foods [5,10]. The GC-O method can be considered an instrumental and also a sensory analysis, describing the stimulus, and evaluating and measuring impressions of the judges [5,10,12].

GC is a high-resolution technique that, in addition to providing information about volatile compounds, relates information to the identification and quantification of compounds in the perception of aroma. Qualitative and quantitative information are obtained from the use of a mass spectrometry detector, which can be a highly selective technique for the analyte of interest. This technique is the most widely used method for identifying chemical compounds in aroma analysis. After separation in the chromatographic column, the molecules in the gaseous state are ionized to normally positive ions. These ions, in turn, are accelerated by an electric field and expelled in the direction of the analyzer tube, where they undergo the action of a magnetic field, perpendicular to the direction of propagation. The tube is kept under a high vacuum (~5–10 Pa) to prevent the ions from being deflective by collision with residual gas molecules. The magnetic field deflects the ions in the direction of the detector, which is at the end of the tube. The ions are separated according to the ratio between their masses and their electrical charges, *m*/*z*. If all charges are +1, then *m*/*z* will be numerically equal to mass. The heavier ions do not undergo significant deflection, while the lighter ions are quite deflective, and both do not reach the detector. The mass spectrum is obtained by varying the intensity of the magnetic field, and the characteristics of the mass spectra are used to identify the compounds. The mass/charge ratio obtained can be transformed into information from studies on the fragmentation mechanism of the compounds and also compared to standard spectra, stored in the computer’s memory [9].

According to de-la-Fuente-Blanco and Ferreira [32], the disadvantages and difficulties associated with the technique are related to overwork. The definition of the hierarchy of compounds is only possible after all odor activity values have been estimated (all odorants must be identified and quantified). In addition, it is found that in some cases it is difficult to determine the end of an odor zone, which can be affected using the sniff port design (delivery of the odorants) [33]. In the same sense, difficulties are verified about the number of judges, since the procedure can be time-consuming [10]. Despite the difficulties inherent in any instrumental analysis, the technique has advantages and provides precise information essential for the aroma determination of fruits.

## 3. Techniques to Evaluate the Odoriferous Importance of Chemical Compounds

In sensory analysis, the judges constitute the sensory panel, with a well-planned and defined number of participants, since it is known that there are differences between the limits of detection and the response criteria for each individual. These differences have been attributed to age, response time to the stimulus, and experience with experimental procedures. A group of well-trained individuals is a prerequisite for a reliable GC-O analysis [10]. The judges who constitute the sensory panel of the GC-O method when inhaling the gas that flows from the chromatograph must be able to identify odors and classify them in order of strength or intensity [28].

Considering that GC-O is a method for knowing the odor zones, additional work must be done to identify the role of these odor zones in the food aroma. The perception of odors is dependent on the characteristics of the compounds, and some may be in high concentrations and be poor in active odor, while others may be in trace amounts and be important contributors to the aroma. Most of the methods used to study aromas aim to classify the volatile compounds detected in order of sensory importance. The three main methods used are the detection limit, the detection frequency, and the perceived intensity. The odorant perception threshold indicates the minimum concentration for a compound to be detected or recognized. On the other hand, detection frequency and intensity are characteristic of each judge who participate in the sensory analysis [35].

One of the most common and widely used odor detection limit (threshold) techniques is aroma extraction dilution analysis (AEDA), a relatively simple approach that provides qualitative data on the intensity of aromas. AEDA is based on the maximum number of dilutions in which the odor is still perceived by the judge, with a dilution factor being calculated for each odorous compound. The greater the dilution factor, the greater the odoriferous importance of the compound for the food [36,37].

AEDA has been applied together with GC-O (AEDA/GC-O) in several studies for the detection of the most different aromas in fruits (Table 1). Amanpour et al. [38] used AEDA to identify the key compounds of fresh and roasted terebinth fruits for the first time, determining α-pinene and β-myrcene as the most potent aroma-active compounds. Cuadrado-Silva et al. [39] applied AEDA to identify the aroma compounds and precursors of sour guava (*Psidium friedrichsthalianum* Nied.) using the solvent-assisted flavor evaporation (SAFE) method for the isolation of volatile compounds. These authors have found that ethyl butanoate, (Z)-3-hexenal, and ethyl hexanoate were the key aroma compounds in sour guava. Some sulphur compounds, such as dimethyl disulphide and 2-methyl-1,3-dithiolane, were reported as relevant odorants to guava’s flavor. SAFE distillation is carried out under high vacuum and successfully recovers most of the volatiles, without causing the formation of artifacts or conditions that could alter the key volatile compounds of the fruit; therefore, it is one of the most used volatile extraction and isolation methods in conjunction with AEDA [40]. Lu et al. [41] identified 56 volatile compounds in Ningxia goji berries (*Lycium barbarum* L.) using AEDA/GC-O and found that hexanal and (E)-2-hexenal accounted for 70–94% of the total volatiles. Yan et al. [42] determined with AEDA/GC-O the pre- and post-harvest aroma profiles in the peel and pulp of “Honeycrisp” apples (*Malus* *×* *domestica*). These authors revealed that the peel shows more sesquiterpenoids, aldehydes, and esters than the pulp, in quantity and variety. Hexyl 2-methylbutyrate, α-farnesene, 1,3-octanediol, and hexanal are some of the most potent odorant compounds in “Honeycrisp” apples.

An extremely important parameter based on the odor detection limit is odor activity value (OAV) that is calculated using the ratio between the concentration of an individual component and its perception threshold [36]. Aromatic compounds with OAV > 1 have concentrations in the sample higher than their detection limits and can be considered to contribute to the fruit aroma [43]. Zhu et al. [44] calculated the OAV to determine the key aroma compounds in mulberry fruits using threshold values taken from information available in the literature (Table 1). These authors revealed that benzaldehyde, ethyl butanoate, and (E)-2-nonenal were present in the three samples of mulberry fruits with much higher OAVs than the other compounds identified (about 40, on average).

Bonneau et al. [45] used the OAV values to identify aroma contributors in fresh and dried mangos (*Mangifera indica* L. cv. Kent), comparing experimental OAVs with those reported in the literature for key compounds in mangoes. These authors revealed that dried mangos show about 15 more volatile compounds identified than fresh mangoes. Limonene, β-myrcene, δ-3-carene, and β-caryophyllene were some of the compounds identified with higher OAV values in both fruits. Whereas hexanal and heptanal showed high OAVs only in dried mangoes, mesifuran appeared with a high OAV value only for fresh mangoes.

The OSME technique, considered one of the most suitable approaches to assess the odorous importance of volatile compounds, is also used for the evaluation of aroma compounds and can measure not only the intensity but also the rate and duration of the aroma (Table 1) [46,47]. In this technique, the determination occurs by associating the chromatographic peak and the response intensity of the volatile compound in the overall aroma of the fruit by a trained and experienced sensory panel. The intensity is measured on a scale of 1 to 3, where 1 is the value attributed to low intensity, 2 is the value attributed to medium intensity, and 3 is the value attributed to high intensity. Unlike AEDA, which is based on odor detection thresholds, OSME is based on Stevens’s power law, a physicochemical law that relates the intensity of the perceived aroma with its concentration in the fruit [48], thus providing quantitative intensity data.

Liu et al. [49] used SPME-GC-MS-OSME to characterize aromatic compounds in different mango fruits (*Mangifera indica* L.) with 10 panelists extensively trained to identify eight sensory characteristics, such as “general aroma,” “tropical fruit,” and “citrus.” Janzantti and Monteiro [16] applied headspace (HS) coupled with GC-MS-O to analyze the sensory acceptance of passion fruit during maturation with 4 trained judges. These authors have revealed that the odor intensity (especially of butyl acetate and alpha-terpineol) and the sensory acceptance were higher during ripeness, which indicates that the conventional aroma of passion fruit is completely reached when the fruit is in its full stage of maturation. OSME was also applied by Ferreira et al. [50] to characterize compounds with an active odor of gabiroba fruits (*Campomanesia xanthocarpa* O. Berg). These authors revealed that from the 79 compounds identified, 39 presented odor zones detected. (E)-2-hexenal, ethanol, ethyl hexanoate, hexanal, ethyl butanoate, and hexanoic acid were the major components, while 4-hydroxy-2,5-dimethyl-2(3H)-furanone, and 1-penten-3-ol, even though not usually present, were some of the compounds related to the aroma of gabiroba.

**Table 1:** Most recent studies (2015–2021) with different techniques for the characterization and evaluation of odoriferous compounds in fruits.

Fruit	Volatile Extraction Technique	Chromatographic Detection	Classification Technique for Odoriferous Compounds	Reference
Yellow tamarillo	SAFE distillation	GC-MS-O	AEDA	García et al. [51]
Hardy kiwi	SAFE distillation	GC-MS-O	AEDA	Lindhorst and Steinhaus [52]
Cherimoya	SPME	GC-MS/GC-FID-O	AEDA	Pino and Roncal [53]
Sour guava	SAFE distillation	GC-FID-O	AEDA	Cuadrado-Silva et al. [39]
Ningxia goji berries	SPME	GC-MS-O	AEDA	Lu et al. [41]
*Terebinth*	SAFE distillation	GC-MS-O	AEDA	Amanpour et al. [38]
Lucuma	SAFE distillation	GC-MS-O	AEDA	Inga et al. [54]
“Honeycrisp” apple	Liquid extraction	GC-MS-O	AEDA	Yan et al. [42]
Cascara	SAFE distillation	GC-MS/GC-FID-O	AEDA	Pua et al. [55]
Mango	SAFE distillation	GC-MS	OAV	Bonneau et al. [45]
Orange	HS-SPME	GC-MS	OAV	Rodríguez et al. [56]
Mulberry	SPME	GC-MS/GC-FID-O	OAV	Zhu et al. [44]
Peach	SPME	GC-MS/GC-FID-O	OAV	Zhuand and Xiao [57]
Gabiroba	HS-SPME	GC-MS-O	OSME	Ferreira et al. [50]
Passion fruit	HS	GC-MS-O	OSME	Janzantti and Monteiro [16]
Mango	SPE	GC-MS-O	OSME	Liu et al. [50]
Bayberry	HS-SPME	GC-MS-O	-	Cheng et al. [58]
Tomato	SPME-PFPD	GC-MS-O	-	Du et al. [59]
Black velvet tamarind	SAFE distillation	GC-MS-O	OAV	Lasekan and See [60]
Bayberry	SPME	GC-MS-O	-	Cheng et al. [61]
Strawberry	HS-SPME	GC-MS	-	Li et al. [62]
Murici, bacuri, and sapodilla	HSSE	GC-MS		Uekane et al. [63]
Quince	SPME	GC-MS-O		Choi et al. [64]
Omija	HSSE	GC-MS		Kim et al. [65]
Watermelon	SPME	GC-MS-O		Mendoza-Enano et al. [66]
Pistachio	HS-SPME	GC-MS		Şahanand Bozkurt [67]
Blueberries	Liquid extraction	GC-QTOF-MS		Yuan et al. [68]

## 4. Studies of Aroma Composition of Fruits

Fruit can contain 100 different types of volatile compounds at concentrations normally greater than 30 ppm, and these differ according to its stage of maturity, the type of cultivar, the form of cultivation, its chemical composition (amount of carbohydrates, proteins, and lipids, for example), and the fruit sample (that is, whether the fruit is intact, cut into slices, or in the form of homogenized purees). The combination of these volatiles, their concentration, and their limit of perception in the complex mixture establish the aroma, which is particular to each fruit, although many of them share the same aromatic characteristics [5,6,69,70].

The aromatic compounds of fruits of greater odoriferous importance are derived from carbohydrates, lipids, phenolic compounds, and mono- and sesquiterpenes, among others. They can be classified as primary or secondary volatile compounds according to their origin (present in the tissue of the intact fruit or produced by the rupture of the tissue in an injury, respectively), which influences the final interpretation of the fruit’s aroma. Although numerous compounds are identified as volatile compounds in fresh fruits, not all of them have a direct impact on the fruit’s aroma: their quantitative abundance and perception thresholds must be extensively evaluated to determine the importance of each precursor in the final fruit aroma [71,72].

Therefore, many studies have been carried out in an attempt to predict aroma compounds using gas chromatography and mass spectrometry (GC-MS) [56,62,63,67,68,73]. The information obtained using GC-MS is interesting; however, it is incomplete, since it is necessary to identify the compounds that are important for the aroma [74]. Thus, the use of GC combined with olfactometry (GC-MS-O) adds information to the methods previously implemented and used for this purpose. Among the information that can be obtained through olfactometry are the intensity, frequency, and description of the compounds separated by the chromatographic column in the gas chromatograph, according to the techniques described in item 3.

**Figure 3:**
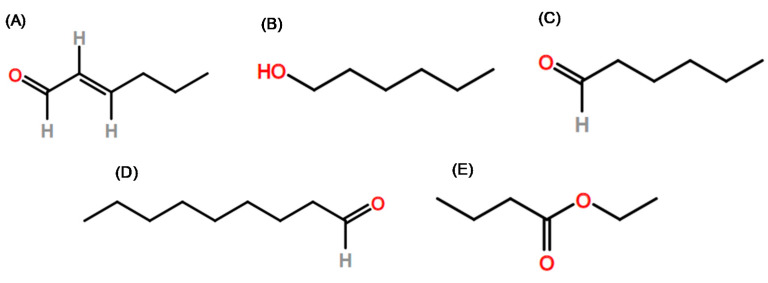
Molecular structure of (**A**) (E)-2-hexenal, (**B**) 1-hexanol, (**C**) hexanal, (**D**) nonanal, and (**E**) ethyl butanoate.

Table 1 provides a list of studies from the past decades involving the identification of aroma compounds using GC-MS-O for fruits, being for many of them the pioneer studies in this field of research, which allowed the volatile compounds responsible for the aroma of some fruits to be unprecedentedly studied. Table 2 demonstrates which compounds are most identified in fruits using GC-MS-O. The odor compounds identified using GC-MS-O that contribute most to the aroma in different fruit species (≥4 species of fruit) are from the classes of alcohols ((E)-2-hexenal, 1-hexanol, and hexanal), aldehydes (nonanal), and esters (ethyl butanoate, Figure 3). The production of these compounds presents biosynthesis directly related to the enzymatic oxidative degradation of unsaturated fatty acids such as linolenic acid via 13-lipoxygenase [54]. The production of aldehydes occurs through the metabolism of fatty acids and through the activity of lipoxygenases (LOX), and their compounds can be reduced to alcohols and, thereby, can be transformed into their corresponding esters [50,75,76]. Although the literature associates the presence of aldehydes with immature fruits [77,78], Egea et al. [79] found aldehyde compounds ((Z)-3-hexenal and hexanal) in strawberry and lemon guava and associated these chemical compounds with green descriptors on GC-O analysis.

## 5. Final Considerations

Gas chromatography-olfactometry (GC-O) has been presented in recent years as an analytical technique that is extremely useful in the identification of precursor compounds of fruit aroma. This technique allows the separation of the compounds through a chromatographic column, followed by their description by judges who are part of a sensory panel, who identify the intensity and frequency of these compounds. Its importance is undoubted, since the knowledge of the components that are part of the sensory attributes allows to evaluate the quality of fruits, with a direct influence on their acceptance or refusal by consumers. However, as it is a recent and extremely broad field of research, there is still much to be done to cover a greater number of fruits, such as evaluating the compounds found by GC-MS for their genetic origin and correlating them with their real implication on the active aroma of fruits.

The results obtained using the GC-MS-O technique demonstrate its importance for the determination of fruit active aroma and for detailing their parameters of intensity, frequency, and description. With this information, many advances can be made in relation to the origin of the fruit, its stage of ripeness, its quality, and its acceptance or refusal by consumers, which reinforces the importance of this technique.

## Figures and Tables

**Table 2:** The five most important chemical compounds found using gas chromatography–olfactometry of fruit aroma (per column) related by the literature (2015–2021).

Chemical Compounds	Fruits																		
	Bayberry ^1^	Bayberry ^2^	Cherimoya ^3^	Cascara ^4^	Gabiroba ^5^	Hardy Kiwi ^6^	“Honeycrisp” Apple ^7^	Lucuma ^8^	Mango ^9^	Mulberry ^10^	Ningxia Goji Berries ^11^	Passion ^12^	Peach ^13^	Quince ^14^	Sour Guava ^15^	Tamarind ^16^	Terebinth ^17^	Tomato ^18^	Watermelon ^19^
(Z)-3-hexenal						X													
(E)-2-hexenal	X	X			X		X	X			X		X						X
(E)-2-Nonenal		X								X									
(E)-2-Octenal																		X	X
(E,E)-2,4-Hexadienal		X																	
(Z)-3-hexen-1-ol													X						
(Z)-3-hexenal								X							X			X	
(Z)-6-nonenal																			X
(Z)-β-Ocimene								X									X		
1,3-octanediol							X												
1,8-cineol						X													
1-hexanol	X								X	X	X								
1-Octen-3-one						X												X	
2,3-butanediol																			
2,3-butanedione								X											
2-methyl-1,3-dithiolane															X				
3-mercaptohexanol													X						
3-methylbutyl 3-methylbutanoate			X																
3-methylbutyl butanoate			X																
3-octanone																			X
4-Hydroxy-2,5-dimethyl-3(2H)-furanone																X			
4-Oxoisophorone				X															
6-Methyl-5-hepten-2-one																		X	X
benzaldehyde										X									
butyl butanoate			X																
cinnamyl acetate																X			
cis-4,5-epoxy-(2E)-undec-2-enal						X													
cis-linalool oxide																X			
Citral																		X	
diethyl carbonate												X							
dimethyl disulphide															X				
ethanol					X														
ethyl (E)-2-butenoate														X					
ethyl 2-methylbutenoate														X					
ethyl 2-methylpropanoate														X					
ethyl butanoate					X					X		X			X				
ethyl cis-3-hexenoate												X							
ethyl hexanoate					X							X			X				
geranyl acetone																X			
Guaiacol				X															
hexanal	X	X			X		X		X	X	X		X						
hexyl 2-methylbutyrate							X												
isoamylol											X								
isocaryophyllene	X																		
Linalool																	X		
methional								X						X					
methyl 2-methylbutanoate			X																
nonanal	X	X									X		X			X			
Ocimene																	X		
p-cymene									X										
propyl acetate												X							
Sotolone				X															
terpinen-4-ol																			
terpinolene									X										
trans-4,5-epoxy-(2E)-dec-2-enal						X													
α-farnesene							X												
α-Pinene			X														X		
β-Damascenone				X															
β-Ionone				X															
β-Myrcene																	X		
γ-decalactone														X					
γ-terpinene									X										

^1^ Cheng, Chen, Chen, Wu, Liu and Ye [58]; ^2^ Cheng, Chen, Chen, Xia, Liu and Ye [61]; ^3^ Pino and Roncal [53]; ^4^ Pua, Choo, Goh, Liu, Cornuz, Ee, Sun, Lassabliere and Yu [55]; ^5^ Ferreira, Garruti, Barin, Cichoski and Wagner [50]; ^6^ Lindhorst and Steinhaus [52]; ^7^ Yan, Shi, Ren, Tao, Ma, Li, Liu and Liu [42]; ^8^ Inga, García, Aguilar-Galvez, Campos and Osorio [54]; ^9^ Liu, An, Su, Yu, Wu, Xiao and Xu [49]; ^10^ Zhu, Wang, Xiao and Niu [44]; ^11^ Lu, Li, Quan, An, Zhao and Xi [41]; ^12^ Janzantti and Monteiro [16]; ^13^ Zhu and Xiao [57]; ^14^ Choi, Lee, Lee and Kim [64]; ^15^ Cuadrado-Silva, Pozo-Bayón and Osorio [39]; ^16^ Lasekan and See [60]; ^17^ Amanpour, Guclu, Kelebek and Selli [38]; ^18^ Du, Song, Baldwin and Rouseff [59]; ^19^ Mendoza-Enano, Stanley and Frank [66].

## Data Availability

Not applicable.

## Sample Availability

Samples of the compounds are available from the authors.
